# Effect of the EM Bokashi® Multimicrobial Probiotic Preparation on the Non-specific Immune Response in Pigs

**DOI:** 10.1007/s12602-018-9460-5

**Published:** 2018-09-05

**Authors:** Ewa Laskowska, Łukasz Sebastian Jarosz, Zbigniew Grądzki

**Affiliations:** Department of Epizootiology and Clinic of Infectious Diseases, Faculty of Veterinary Medicine, University of Life Sciences in Lublin, Głęboka 30, 20-612 Lublin, Poland

**Keywords:** Multimicrobial probiotic preparation, Phagocytic activity, Oxidative burst, Cytokine, Lysozyme, Pigs

## Abstract

The aim of the study was to determine the effect of EM Bokashi® on the phagocytic activity of monocytes and granulocytes, oxidative burst, SWC3, and CD11b + CD18+ expression on monocytes and granulocytes, and the serum concentration of cytokine and lysozyme in pig. 60 Sixty female piglets were divided into two groups: I – control and II – experimental. For the experimental group, a probiotic in the form of the preparation EM Bokashi® was added to the basal feed. Flow cytometry was used to determine selected non-specific immune response parameters, intracellular production of hydrogen peroxide by peripheral granulocytes and monocytes, and surface particles in peripheral blood. The EM Bokashi® preparation used in the study was found to increase phagocytic activity mainly in monocytes, with an increased percentage of phagocytic cells in the experimental group. The highest serum lysozyme concentration in the piglets in the experimental group (2.89 mg/dl), was noted on day 42 of the study. In the group of pigs receiving EM Bokashi®, the percentage of phagocytic cells with SWC3 (monocyte/granulocyte) expression was statistically significantly higher than in the control. The increase in the number of cells with SWC3 (monocyte/granulocyte) expression in the peripheral circulation in combination with the greater capacity of the cells for phagocytosis and respiratory burst confirms that the non-specific immune response was modulated in the pigs supplemented with EM Bokashi®.

## Introduction

Modern pig farming requires a variety of technological measures to reduce economic losses resulting from intensified production and the associated deterioration in the health of pigs [1]. One of the most important factors affecting the efficiency of pig production is proper nutrition [2]. Improving the utilization of nutrients contained in feed by increasing its digestibility [3] while maintaining homeostasis within the gastrointestinal tract is currently the greatest challenge for modern production systems [4]. For many years, one means of increasing the growth rate of pigs and limiting the development of infections by pathogenic microorganisms was the use of antibiotics as feed additives (antibiotic growth promoters—AGP) [5]. The ban on the use of AGPs introduced in 2006 necessitated the search for alternative feed components that would ensure good health and high production indicators in pigs. The use of probiotics as feed additives for pigs has proven to be a solution that meets the expectations of producers, veterinarians, and consumers [6, 7].

The probiotics most frequently used in pig feeding are based mainly on bacteria of the genera *Lactobacillus*, *Bifidobacterium*, and *Enterococcus* [8]. They have been shown to maintain the microbial balance within the gut, stimulate enterocyte development, regulate gastrointestinal motility, improve digestion and absorption processes, and participate in the production of organic acids and metabolites that neutralize bacterial toxins [9–12]. However, the main effect of probiotics in the gastrointestinal tract of pigs is the prevention of colonization of the mucosa by pathogenic microbes [9, 11, 12]. The mechanism of this effect of probiotics is based on competition with pathogens for adhesion sites on enterocytes and stimulation of local and systemic immune mechanisms in the host [13, 14]. Probiotics also have an immunomodulatory effect, although its exact mechanism is not yet fully understood. The immunomodulatory potential of probiotics is thought to involve maintaining the Th1/Th2 balance in the body in cooperation with Th17 cells and regulatory T (Treg) cells [15]. Probiotics also stimulate immunocompetent cells to produce cytokines that enhance, reduce, or regulate the systemic and local immune response [16, 17]. The individual bacterial strains contained in probiotics have a varied effect on immunity [15]. Some strains have the capacity to activate various types of Th cells, while others activate only one type. For example, *L. casei*, *L. gasseri*, *L. johnsonii*, *L. reuteri*, and *L. lactis* induce a Th1 response [18, 19], *L. rhamnosus* induces a Th1 and a Th17 response [20], while *L. reuteri* and *L. salivarius* stimulate production of IL-10, activating a Th2-type response [18, 21]. In addition, some microbial strains not only affect humoral and cellular immune mechanisms, but also stimulate a nonspecific immune response, increasing the activity of phagocytic cells and NK cells [22], which are the first line of defense against pathogens [23]. Phagocytic cells, monocytes, macrophages, polymorphonuclear cells (PMNs), and NK cells are induced by various stimuli and initiate numerous intracellular reactions, such as cytokine secretion and the production of reactive oxygen and nitrogen species [24]. The use of probiotics as feed additives may be one of the stimuli that effectively stimulate a nonspecific immune response in pigs [25]. Previous studies, conducted mainly in humans, have shown that the immunomodulatory effect expressed as an increase in the activity of phagocytic cells varies depending on the species and strain of bacteria in the probiotic [26], the viability of the bacteria [27], and the dose of the preparation used [28].

Furthermore, an increase in phagocytic activity has been observed in the case of probiotics based on single bacterial strains as well as multiple strains [29, 30]. According to Cho et al. [31], the immunomodulatory effect of microbial strains used as probiotics in feeding pigs consists in induction of cytokines, an increase in local antibody synthesis, induction of interferon and NK cell production, and especially in an increase in macrophage activity [22].

Probiotic preparations based on effective microorganisms (EM) are increasingly used in feeding pigs [8, 9]. The term “EM” is often used by manufacturers in the names of products containing microbial strains which are not subjected to any technological processing, as an indicator of the high-quality such products. Preparations containing effective microorganisms, which are combinations of 70 to 80 strains of useful microbes, are widely used in numerous fields associated with agriculture and livestock farming [32, 33]. The beneficial effect of EM on animals is manifested as increased daily weight gain, improved feed digestibility, reduced mortality, and improved health [34–36]. One of the EM-based preparations used in pig diets is EM Bokashi^®^. It contains microbial strains which are not subjected to any technological processing. Preparations with this type of composition have previously been shown to be an innovative solution with the ability to stimulate systemic immune mechanisms [37, 38]. However, the mechanisms of modulation of the nonspecific immune response in pigs by EM Bokashi^®^ are as yet unknown. No studies have been conducted on the use of this preparation in pigs.

The aim of the study was to determine the effect of EM Bokashi^®^ on the phagocytic activity of monocytes and granulocytes, oxidative burst, SWC3, and CD11b^+^CD18^+^ expression on monocytes and granulocytes, and the serum concentration of cytokine and lysozyme in pigs from birth to the end of the fattening period.

## Materials and Methods

### Experimental Animals and Use of the EM Bokashi^®^ Preparation

All procedures used during the research were approved by the Local Ethics Committee for Animal Testing at the University of Life Sciences in Lublin, Poland (approval no. 55/2013, 15 October 2013).

The study was conducted on a private pig breeding farm with a closed production cycle. The farm kept 150 sows aged 2–4 years with a body weight of 150–200 kg. The farm had the status of free of certain infectious diseases, including porcine reproductive and respiratory syndrome (PRRS), pleuropneumonia, mycoplasmosis, and streptococcosis, as confirmed by screening tests performed at the National Veterinary Research Institute in Puławy. Pregnant sows were regularly vaccinated against parvovirus and erysipeloid of Rosenbach.

The study was conducted on 60 female piglets (Polish Large White x Polish Landrace), all born at the same time and on the same farm, weighing 1.10–1.30 kg at birth. The piglets were divided into two groups of 30 piglets each: group I–control and group II–experimental. Each piglet from the control and experimental groups was marked with an ear tag with an individual number. The piglets stayed with their mothers until their 28th day of life. Then, they were weaned from their mothers and moved to group pens with 10 piglets each, where they remained until the end of the experiment. The size, temperature, humidity, and hygiene conditions of the pens were identical for the two groups.

The newborn piglets had constant access to fresh drinking water. From the 7th day of life, they had constant access to complete compound feed for piglets (see Table 1). Weaners and fatteners were fed ad libitum from automatic feeders with the complete compound feeds used on the farm, with constant access to water from automatic drinkers, see Table 1. The diets were formulated to meet or exceed the nutrient requirement recommendations of the NRC (1998) [29]. All pigs were fed the same basal diet during the experiment. No antibiotic growth promoters or antibiotic treatment were used during the entire experimental period.

**Table 1:** Ingredients and nutritive value of the pigs diets (as-fed basis)

Ingredient (g)	Starter^a^≥ 12 kg	Grower 12–30 kg	Finisher 30–110/115 kg
Barley	–	37.0	39.5
Wheat	–	39.5	39.0
Corn/gelatinized corn	41.0/3.2	–	–
Soybean meal over 46% HP	35.0	10.0	14.0
Fermented rapeseed meal	–	2.0	4.0
Fermented soybean meal	2.0	2.0	2.5
Soybean oil	15.0	2.0	1.0
Starch	1.8–2.0	–	–
Complementary feed 4% MPU START 820115	2.0	4.00	–
Concentrate MHP	–	3.0	–
Propionic acid	–	0.5	–
Bicalcium phosphate/limestone	0.46/0.30	–	–
Vitamin/mineral suplement	0.1/0.33	–	–
L-lysine HCL 78%/DL-methionine 98%	0.076/0.09	–	–
Nutritive value			
Metabolizable energy, MJ/kg	13.9	13.3	13.5
Crude protein, g	201	178	174
Dry matter, g	–	827	850
Lysine, g	15.45	12.3	10 > 3
Methionine,g	3.72	4.47	3.06
Methionine + cystine, g	6.56	8.15	6.48
Threonine, g	7.35	7.74	6.54
Tryptophan, g	2.22	1.95	1.99
Digestible lysine, g	12.29	1.70	3.22
Total phosphorus, g	4.52	5.25	6.01
Digestible phosphorus, g	–	4.28	3.74
Calcium, g	8.26	8.27	6.49
Sodium, g	–	2.33	1.67
Fiber, g	36.0	36.1	40.0
Raw fat, g	–	39.3	28.6
Vitamin A, IU	–	16,000.0	10,500.0
Vitamin D_3_, IU	–	2000.0	2000.0
Vitamin E, mg	–	115	115

^a^Commercial product without probiotic, data in the table provided by the manufacturer

The control and experimental groups were housed in separate pig houses, with identical building and environmental conditions, in order to prevent probiotic (EM Bokashi®) cross-contamination. The pigs in group I, the control, were fed from the 7th day of life to the end of the experiment on standard feed without a probiotic supplement. For the pigs in group II, the experimental group, a probiotic in the form of the preparation EM Bokashi^®^, was added to the basal feed in the amount of 10 kg/t of feed, from the 7th day of life to the end of the experiment. The probiotic EM Bokashi^®^ used in the experiment is manufactured by Greenland Technologia EM, Janowiec, Poland, and contains a mixture of microorganisms, which were described by Laskowska et al. [38]. The feed for the control and experimental groups was prepared daily for the duration of the experiment. Throughout the experimental period, the EM Bokashi® preparation was tested once a month in the national reference laboratory of the Department of Hygiene of Animal Feedingstuffs of the National Veterinary Research Institute in Puławy. The viability of probiotic bacterial cells and their content per gram of the product (CFU/g) was tested as described by Weese and Martin [39], to ensure that the experimental conditions were the same throughout the experiment. Furthermore, the company manufacturing the EM Bokashi® preparation evaluated the viability of probiotic bacterial cells and their content per gram of the product in their laboratory, thus guaranteeing that the product used in the experimental group of sows was of the same qualitative composition throughout the experiment.

### Clinical Signs in the Animals

Throughout the experiment, the pigs were under clinical observation, with special attention paid to their activity, appetite, respiratory symptoms, and the occurrence of digestive disorders in the form of diarrhea (Table 2).

**Table 2:** Effect of the use of effective microorganisms on pig health

Day of life	Group I	Group II
0	–	–
7	Cough *n* = 8	–
14	Diarrhea *n* = 8	Diarrhea *n* = 5
	Decreased appetite *n* = 6	Decreased appetite *n* = 4
21	–	–
28	Diarrhea *n* = 12	Diarrhea *n* = 6
	Sneezing *n* = 21	Sneezing *n* = 11
	Decreased appetite *n* = 7	Decreased appetite *n* = 4
35	Diarrhea *n* = 9	Cough *n* = 6
	Cough *n* = 14	Sneezing *n* = 9
	Sneezing *n* = 18	
	Decreased appetite *n* = 8	
42	Cough *n* = 13	–
	Sneezing *n* = 22	
63	Diarrhea *n* = 7	–
	Cough *n* = 14	
	Sneezing *n* = 18	
	Decreased appetite *n* = 8	
70	Cough *n* = 10	Cough *n* = 8
98	–	–
126	–	–
154	–	–

### Blood Sample Collection

The material for the study consisted of peripheral blood collected from the external jugular vein into Vacuette vacuum tubes containing heparin as an anticoagulant (Medlab Products, Poland) EDTA-K_2_ tubes (Medlab Products), and serum separation tubes with a clot activator (Medlab Products, Poland). Blood samples were collected from all pigs in groups I and II at 0, 7, 14, 21, 28, 35, 42, 63, 70, 98, 126, and 154 days after birth. The samples were transported chilled to the laboratory at + 4 to + 8 °C for no longer than 1 h. The blood collected into EDTA-K_2_ and heparin tubes was used for cytometric analysis immediately after the material was delivered to the laboratory. The blood collected into serum separation tubes with a clot activator was centrifuged at room temperature (20–22 °C) for 15 min at 1000*g*, and the serum obtained was apportioned and stored for further study at − 80 °C.

### Determination of Non-specific Immune Response Parameters

Flow cytometry was used to determine selected non-specific immune response parameters. Blood samples were tested in a BD FACSVerse™ flow cytometer (Becton Dickinson, Poland). Green fluorescence (from FITC) was detected on the FL1 channel (527/32 nm band pass filter) and orange fluorescence (from RPE) was detected on the FL2 channel (586/42 nm band pass filter). Cells were analyzed at up to 20,000 events. Electronic compensation was used to eliminate residual spectral overlaps between individual fluorochromes. Flow cytometry was repeated three times for each sample and compared for repeatability.

### Assay of SWC3 and CD11b^+^CD18^+^ Surface Particles in Peripheral Blood

**Fig. 1:**
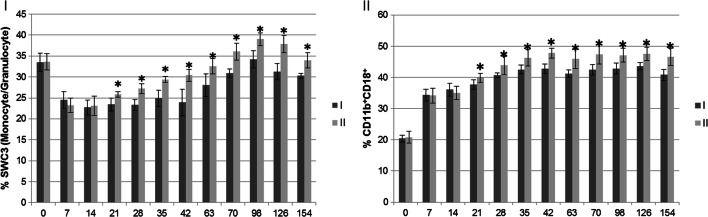
Percentage of subpopulation SWC3 (monocyte/granulocyte)-I and CD11b^+^CD18^+^-II in the peripheral blood of pigs from groups I and II. I-control group, II-experimental group. Values are expressed as the mean and standard deviation (α^+^/-SD). Asterisks indicate a significant increase in the parameter (*p < 0.05) between control and experimental group

The following fluorochrome-labeled monoclonal antibodies for the surface molecules of pig cells were used in the cytometric tests: SWC3 (mouse anti-porcine monocyte/granulocyte)/FITC (clone 74-22-15, Beckman Coulter, Inc., Fullerton, USA), CD11b (clone CA16.3E10, Bio-Rad, Puchheim, Germany), secondary antibodies for CD11b–Rabbit F(ab′)2 anti-Mouse IgG:FITC (STAR9b, Bio-Rad, Puchheim, Germany), and CD18/RPE (clone YFC118.3 Bio-Rad, Puchheim, Germany). The antibodies used in the negative isotopic control were mouse IgG1 conjugated with FITC and rat IgG2b conjugated with RPE. The method described by Stabel et al. [40] was used to assess the immunophenotype of the pig blood cells. See Fig. 1.

### Assessment of Phagocytosis

**Fig. 2:**
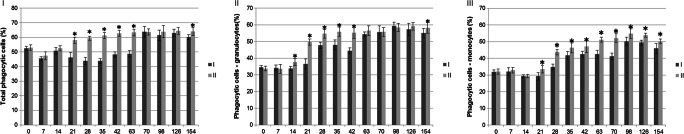
Assessment of phagocytosis: I–percentage (%) of total phagocytic cells, II–percentage (%) of phagocytic cells–granulocytes, III–percentage (%) of phagocytic cells–monocytes in pigs. I–control group, II-experimental group. Values are expressed as the mean and standard deviation (α^+^/-SD). Asterisks indicate a significant increase in the parameter (*p < 0.05) between control and experimental group

Phagocytosis was measured in whole blood with the Phagotest kit (ORPEGEN Pharma, Heidelberg, Germany). Blood samples were prepared according to the test manufacturer’s recommendations. The individual determinations were carried out using a test and control sample with a 100-μl volume of whole blood, which was first incubated in an ice bath for 10 min. After incubation, 20 μl of FITC-labeled *E. coli* suspension was added to both samples. After thorough mixing, the control sample was again placed in an ice bath and the test sample was incubated in a water bath at 37 °C for 10 min. Then, the test sample was transferred to an ice bath to stop the phagocytosis process. Next, 100 μl of chilled quenching solution was added to both samples and the contents were mixed thoroughly. Then, the test and control cell suspensions were washed twice with washing solution, using 3 ml of liquid each time and in each case centrifuging the mixture at 250 g for 5 min at 4 °C and decanting the supernatant. After washing, 2 ml of FACS lysing solution (Becton-Dickinson) diluted 1:10 in distilled water was added to both samples, mixed vigorously, and incubated at room temperature (18–25 °C) for 20 min. The cell suspension was then centrifuged at 250*g* for 5 min, at 4 °C, the supernatant was decanted, and again, the suspension was washed twice with washing solution under the previous conditions. After the last centrifugation and decanting of the supernatant, 200 μl of staining solution for DNA was added to the precipitate, and after thorough mixing, the mixture was incubated in an ice bath for 10 min. Cytometric analysis of the samples prepared in this manner (test and control) was performed using a BD FACSVerse™ flow cytometer (Becton Dickinson, Warsaw, Poland). Phagocytosis was measured quantitatively on the basis of the percentage of phagocytic cells (see Fig. 2) and qualitatively based on the average fluorescence intensity of FITC for monocytes and granulocytes (see Table 3). Phagocytosis in the test and control samples was assessed separately for all phagocytic cells and independently for granulocytes and monocytes.

**Table 3:** Mean fluorescence intensity of peripheral blood phagocytic cells of pigs from groups I and II. Values are expressed as the mean and standard deviation (α+/-SD)

Day	Mean fluorescence intensity in granulocytes		Mean fluorescence intensity in monocytes	
	Group I	Group II	Group I	Group II
0	518.10 ± 92.1	543.60 ± 89.7	211.77 ± 74.1	222.91 ± 83.7
7	479.80 ± 95.4	422.80 ± 101.5	238.16 ± 72.4	234.71 ± 63.1
14	521.16 ± 112.8	495.63 ± 105.3	233.62 ± 52.1	241.12 ± 62.8
21	357.40 ± 101.2	481.14 ± 93.5^a^	218.20 ± 64.5	221.82 ± 59.4
28	397.11 ± 95.4	498.55 ± 99.7^a^	255.68 ± 73.1	295.44 ± 48.9^a^
35	412.17 ± 110.8	511.22 ± 101.3^a^	261.15 ± 68.7	302.16 ± 98.5^a^
42	435.11 ± 99.4	516.84 ± 86.4^a^	268.22 ± 57.3	311.18 ± 84.3^a^
63	520.19 ± 121.3	744.31 ± 124.88^a^	318.14 ± 62.1	378.42 ± 58.6^a^
70	684.22 ± 117.3^a^	521.15 ± 119.1	351.22 ± 63.5	392.19 ± 54.2^a^
98	621.11 ± 108.7^a^	477.25 ± 103.2	372.14 ± 74.8	403.15 ± 61.3^a^
126	576.52 ± 111.4^a^	492.11 ± 95.1	377.81 ± 69.7	384.12 ± 72.1
154	493.28 ± 79.7	471.84 ± 84.2	315.69 ± 73.2	325.11 ± 49.7

^a^Asterisks indicate a significant increase in the parameter (*p < 0.05) between control and experimental group. I-control group, II-experimental group

### Respiratory Burst of Granulocytes and Monocytes

Intracellular production of hydrogen peroxide by peripheral granulocytes and monocytes was assessed by flow cytometry in whole blood using the fluorogenic substrate dihydrorhodamine (DHR) 123 (BurstTest kit, ORPEGEN Pharma, Heidelberg, Germany). Briefly, ice-cold heparinized blood (100 μl) was activated with unlabeled opsonized *Escherichia coli* for 10 min at 37.0 °C. DHR 123 was then added and incubation was continued for another 10 min at 37.0 °C. Erythrocytes were lysed and fixed for 20 min at room temperature. Samples were washed twice with washing solution by centrifugation (5 min, 1200 rpm, 4 °C) and the supernatant was discarded. Finally, 200 μl of propidium iodide (PI–DNA staining solution) was added while the samples were kept on ice in a dark place. The cells were analyzed by flow cytometry using a BD FACSVerse™ flow cytometer (Becton Dickinson, Warsaw, Poland). At least 10,000 events were analyzed (Table 4).

**Table 4:** Granulocyte and monocyte oxidative burst under *E. coli* stimuli of pigs from groups I and II. Values are expressed as the mean and standard deviation (α+/-SD)

Day	Oxidized cells (%)	
	*E. coli* stimulation	
	Group I	Group II
0	48.6 ± 5.3	47.7 ± 6.8
7	45.1 ± 7.2	46.6 ± 5.8
14	46.1 ± 6.3	49.8 ± 7.1
21	59.6 ± 9.2	65.4 ± 9.2^a^
28	64.5 ± 8.1	76.8 ± 10.1^a^
35	72.6 ± 9.5	89.4 ± 11.3^a^
42	64.3 ± 10.2	78.5 ± 8.7^a^
63	56.8 ± 9.1	76.6 ± 7.2^a^
70	62.4 ± 11.2	71.2 ± 6.5^a^
98	73.4 ± 12.3	72.6 ± 11.4
126	79.5 ± 10.8	75.8 ± 10.2
154	56.7 ± 9.7	69.7 ± 8.2^a^

^a^Asterisks indicate a significant increase in the parameter (*p < 0.05) between control and experimental group. I-control group, II-experimental group

### Determination of the Bacteriolytic Activity of Lysozyme in the Peripheral Blood of Pigs

**Fig. 3:**
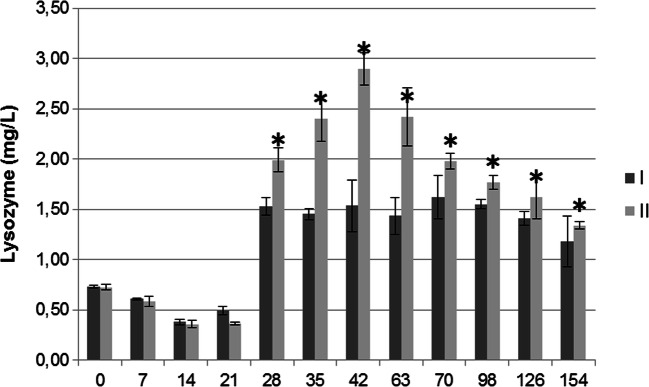
Lysozyme concentration (mg/l) in the peripheral blood of pigs from groups I and II. I–control group, II-experimental group. Values are expressed as the mean and standard deviation (α^+^/-SD). Asterisks indicate a significant increase in the parameter (*p < 0.05) between control and experimental group

The lysozyme concentration in the serum of pigs from groups I and II (Fig. 3) was determined in relation to that of *Micrococcus luteus* (Serva) by the plate method according to Graham, as described by Hankiewicz and Świerczek [41].

### Assay of IL-6, IL-10, and TNF-α in Pig Serum

ELISA kits specific for porcine IL-6, IL-10, and TNF-α (Wuhan Fine Biotech Co., Ltd., East Lake High-tech Development District, Wuhan, Hubei Province, China) were used to determine the cytokine levels in the serum. All procedures were performed according to the manufacturer’s instructions. The absorbance was recorded at 450 nm using an ELISA plate reader (Multiskan RC, Labsystems, Finland). Each sample was tested in three replicates. The results were expressed as mean and standard deviation (±SEM); values of *p* < 0.05 were regarded as significant (Table 5).

**Table 5:** Serum concentrations of IL-6, IL-10, and TNF-α in pigs from groups I and II. Values are expressed as the mean and standard deviation (α+/-SD)

	IL-6		TNF-α		IL-10	
Day	I	II	I	II	I	II
0	10.82 ± 5.43	10.12 ± 6.11	–	–	10.14 ± 6.12	9.06 ± 4.21
7	20.28 ± 8.25	21.11 ± 12.73	28.14 ± 11.55	26.18 ± 9.27	15.21 ± 7.12	16.12 ± 8.73
14	28.34 ± 10.21	27.62 ± 11.89	30.21 ± 17.23	31.11 ± 12.39	18.33 ± 7.82	19.05 ± 10.22
21	38.16 ± 12.87	39.41 ± 16.59	33.16 ± 18.22	35.82 ± 11.72	29.64 ± 10.11	31.82 ± 7.99
28	80.62 ± 18.65	81.89 ± 15.94	37.98 ± 9.91	36.98 ± 9.89	45.75 ± 14.95	45.65 ± 15.25
35	87.25 ± 19.23	86.84 ± 19.22	39.21 ± 11.12	59.64 ± 10.81^a^	46.22 ± 11.25	52.11 ± 12.38
42	96.37 ± 21.84	93.28 ± 21.33	45.54 ± 10.95	87.87 ± 25.53^a^	45.53 ± 18.29	58.59 ± 11.25^a^
63	105.84 ± 17.25	127.83 ± 19.27^a^	43.11 ± 9.72	94.25 ± 10.08^a^	36.24 ± 12.79	69.27 ± 16.31^a^
70	139.57 ± 24.71	148.75 ± 28.06^a^	40.90 ± 12.35	97.94 ± 21.76^a^	38.64 ± 9.28	76.02 ± 11.89^a^
98	111.42 ± 22.11	152.84 ± 21.63^a^	47.28 ± 7.56	105.21 ± 12.86^a^	39.92 ± 11.73	81.14 ± 15.22^a^
126	108.70 ± 16.63	164.20 ± 23.39^a^	57.78 ± 9.34	111.31 ± 14.04^a^	40.47 ± 14.23	89.39 ± 12.74^a^
154	151.14 ± 26.44	248.26 ± 30.43^a^	87.28 ± 10.21	135.70 ± 16.55^a^	38.76 ± 16.21	79.83 ± 10.95^a^

^a^Asterisks indicate a significant increase in the parameter (*p < 0.05) between control and experimental group. I-control group, II-experimental group

### Statistical Analysis

The results were analyzed statistically using Statistica 10.0 PL (StatSoft, Krakow, Poland). The analysis included the arithmetic mean and standard deviation (α ± SD). The significance of differences between means obtained for the control and experimental groups of animals was assessed by the nonparametric Mann-Whitney *U* test, and *p* values of less than 0.05 were considered to indicate statistical significance (Tables 3, 4, 5, and Figs. 1, 2, 3).

## Results

### Effect of the Use of Effective Microorganisms on Pig Health

During the experiment, diarrhea and decreased appetite were observed in some pigs from the control group on days 7, 14, 28, and 63 of the study. Sneezing and coughing were also observed in pigs in the control group on days 28, 35, 42, 63, and 70 days of the study. In some of the piglets in the experimental group, diarrhea and loss of appetite were observed on days 14 and 28, and coughing and sneezing on days 28, 35, and 70. See Table 2.

### Assay of SWC3 (Monocyte/Granulocyte) and CD11b/CD18 Surface Particles in the Peripheral Blood

Statistically significantly higher means for the percentage of the SWC3 subpopulation (monocyte/granulocyte) in the peripheral blood were observed in the pigs from the experimental group from days 21 to 154 of the study compared to the control group (Fig. 1). In comparison to the control group, statistically significantly higher means for the percentage of CD11b^+^CD18^+^ subpopulations in the peripheral blood of pigs from the experimental group were observed between days 21 and 145 of the study (Fig. 1).

### Evaluation of Phagocytosis and Respiratory Burst

A statistically higher percentage of total phagocytic cells in pigs in the experimental group compared to the control group was observed on days 21, 28, 35, 42, 63, and 154 of the study (Fig. 2I). A significantly higher percentage of phagocytic cells–granulocytes in the pigs in the experimental group compared to the control group was observed on days 14, 21, 28, 35, 42, and 154 of the study (Fig. 2II). A statistically significantly higher percentage of phagocytic cells–monocytes in the pigs in the experimental group compared to the control group was observed from days 21 to 154 of the study (Fig. 2III).

On days 21, 28, 35, 42, and 63 of the study, statistically significantly higher mean fluorescence intensity in the granulocytes was observed in the experimental group in comparison to the control group. However, on days 70, 98, 126, and 154 of the study, the mean fluorescence intensity in the granulocytes was statistically significantly higher in the control group than in the experimental group (Table 3). The mean fluorescence intensity in the monocytes was statistically significantly higher on days 28, 35, 42, 63, 70, and 98 in the experimental group than in the control group (Table 3).

In comparison to the control group, statistically significantly higher percentages of oxidized cells after *E. coli* stimulation were observed in the experimental group on days 21 to 70 and on day 154 (Table 4).

### Evaluation of Cytokine and Lysozyme Concentrations in the Peripheral Blood of Pigs

In comparison to the control group, statistically significantly higher serum concentrations of IL-6 were observed in the experimental group between days 63 and 154 of the study. Compared to the control group, statistically significantly higher serum concentrations of TNF-α were observed in the experimental group between days 35 and 154 of the study. Statistically significantly higher serum concentrations of IL-10 were observed in the experimental group between days 42 and 154 of the study in comparison to the control group (Table 5).

Compared to the control group, statistically significantly higher concentrations of lysozyme were observed in the peripheral blood of pigs from the experimental group between days 28 and 145 of the study (Fig. 3).

## Discussion

One of the most important effects of probiotics on the immune response is the promotion of endogeneous host defense mechanisms [25]. In addition to their effect on non-immune intestinal defenses, by stabilizing the intestinal flora, these preparations modulate the host’s systemic and local immune response. Their main effect is immunostimulation, expressed as the induction of cellular and humoral immunity, increased production of immunoglobulins and interferon, activation of macrophages, lymphocytes and NK (natural killer) cells, and regulation of phagocytosis and respiratory burst [42]. These processes help to maintain the Th1/Th2 balance and stimulate the production of specific types of cytokines [43, 44]. Rask et al. and Ho et al. [45, 46] showed that probiotic bacteria stimulate the immune response in a manner specific for a given strain, so that the mechanisms of their immunomodulatory activity are varied. Therefore, to ensure the full spectrum of the beneficial effects of probiotics, the probiotic preparations must contain a combination of many strains of microorganisms [47]. These advantages are found in the EM Bokashi® preparation used in the experiment, composed of a variety of microbial strains [48].

An important function of probiotics is the aforementioned effect on macrophage activity and regulation of phagocytosis and respiratory burst [49, 50]. Monocytes and macrophages are cellular components of the innate immune system which prevent pathogen invasion by releasing cytotoxic molecules, such as reactive oxygen species (ROS), and by secreting proinflammatory cytokines such as TNF-α and IL-8 [51]. These cells play an important role in ensuring homeostasis of the gut environment, acting as ‘immunological sentinels’ [52]. They are able to initiate an inflammatory response in response to infection, and impairment of their function leads to disturbances in immune processes, e.g., in the course of chronic diseases [51]. Cummings et al., 2004 [53] and Reiner, 1994 [54] have shown that phagocytic activity and respiratory burst in phagocytic cells can be regarded as a marker of the influence of probiotics on the nonspecific immune response and the host’s defense capability against pathogenic microbes [53, 54]. The present study showed a statistically significantly higher percentage of phagocytic cells in pigs receiving the EM Bokashi® preparation as compared to the control between days 21 and 63 of the study and on day 154. In the remaining periods of the study, no statistically significant differences were found between the two groups. Similar observations were made by Ren et al. [55] in a study using *L. plantarum* and by Kausahal et al., who assessed the effect of *Lactobacillus acidophilus* and *Bifidobacterium bifidum* on the phagocytic potential of macrophages [56]. Similarly, Rocha-Ramirez et al. [57] showed that probiotic bacteria stimulate the phagocytic activity of macrophages and their microbicidal potential against both extracellular pathogens such as *S. aureus* and *E. coli* and intracellular pathogens such as *S.* typhimurium. Our results have also been confirmed in studies on small mammals, in which the use of probiotics increased the phagocytic activity of macrophages, which facilitates the removal of pathogenic bacteria from the body and is indicative of stimulation of non-specific immune mechanisms [58]. Similarly, in our study, a reduction in gastrointestinal and respiratory symptoms was observed in the group of pigs treated with the EM Bokashi® preparation, most likely linked to stimulation of mechanisms eliminating pathogenic microorganisms through phagocytosis.

Research by Arunachalam et al. [59] and Donnet-Hughes et al. [28] showed that feed supplementation with probiotic bacteria increases the phagocytic capacity mainly of neutrophilic granulocytes, which is accompanied by increased respiratory burst and increased expression of receptors involved in phagocytosis, mainly complement receptor 3 (CR3). In contrast, the EM Bokashi® preparation used in our study was found to increase phagocytic activity mainly in monocytes, with an increased percentage of phagocytic cells in the experimental group between days 21 and 154 of the experiment. In the case of granulocytes, an increased percentage of phagocytic cells in the experimental group was noted only between days 14 and 42 and on days 126 and 154 of the study. The increased percentage of monocytes and granulocytes between days 21 and 63 of the study indicates stimulation of the immune system by the EM Bokashi® preparation during the periods in which the health of pigs is at the greatest risk. During this period, pigs show increased susceptibility to infection, due to stress factors associated with weaning, the formation of new groups of animals, and changes in the diet. The use of EM Bokashi® as a feed supplement during this period benefited the health of the animals by preventing intestinal infections through facilitation of mechanisms eliminating pathogens causing diarrhea.

The mean fluorescence intensity, which is a marker of the phagocytic activity of granulocytes, was higher in the experimental group between days 21 and 63 of the study and in the control group between days 70 and 126. In the case of monocytes, statistically significant differences in the mean fluorescence intensity between the experimental and control groups were demonstrated between days 28 and 98 of the study. The increase in the mean fluorescence intensity at these times in the group of pigs receiving EM Bokashi®, in conjunction with the increase in the phagocytic activity of the cells and in respiratory burst, is indicative of more effective action of phagocytes against microorganisms and more effective elimination of pathogens from the body. The results also indicate increased efficiency of intracellular killing of microbes by macrophages during this period, which increases the overall health of animals. This is important in the period of weaning and decreased passive immunity, in which animals are exposed to environmental infections that increase the incidence rate and mortality rate. The increased potential of the non-specific immune response resulting from the use of the EM Bokashi® preparation reduces morbidity and mortality by more effectively fighting infections.

In the present study, the serum lysozyme concentration increased in the piglets in the experimental group during the period from days 28 to 42, while the highest concentration of this protein, 2.89 mg/dl, was noted on day 42 of the study. On subsequent days, the lysozyme concentration in the experimental group gradually decreased, but on all test days, i.e., from days 42 to 154, it was higher in the pigs in the experimental group than in the control. The increase in the lysozyme concentration in the experimental group between days 28 and 42 of the experiment demonstrates that this time interval is a critical period in young animals in terms of the risk of infection, mainly due to the change in way of life and diet and exposure to environmental microorganisms. The high lysozyme concentration in the pigs receiving the EM Bokashi® preparation was correlated with other immune parameters, i.e., the expression of monocyte/macrophage and CD11b^+^/CD18^+^ receptors. In both cases, the value of the parameters increased, which demonstrates that the bacterial and fungal antigens in the EM Bokashi® preparation stimulated activation of monocytes and macrophages. It is worth noting that in addition to lysozyme, numerous cytokines take part in the stimulation of macrophages, mainly TNF-α [60], which stimulates macrophages and monocytes to produce lysozyme and releases it from neutrophils. This is confirmed by the high TNF-α concentration from days 35 to 154 of the experiment in the pigs receiving EM Bokashi®. On the other hand, macrophages and monocytes stimulated by TNF-α participate in presentation of antigens contained in the probiotic, influencing humoral, and cellular immune mechanisms, as confirmed in previous studies [37, 38]. A high concentration of lysozyme found locally in the gastrointestinal tract has been shown to affect the gut microbiota by inhibiting the development of pathogenic bacteria, mainly *E. coli* [61]. Feed supplementation with probiotics containing microbes such as *Saccharomyces* spp. inhibits the proliferation of pathogenic strains of ETEC in pigs and stimulates lysozyme synthesis, which has similarly been demonstrated in broilers [62].

In our experiment, in the group of pigs receiving EM Bokashi®, between days 21 and 154 of the experiment, the percentage of phagocytic cells with SWC3 (monocyte/granulocyte) expression was statistically significantly higher than in the control. The increase in the number of cells with SWC3 (monocyte/granulocyte) expression in the peripheral circulation in combination with the greater capacity of the cells for phagocytosis and respiratory burst confirms that the non-specific immune response was modulated in the pigs whose feed was supplemented with EM Bokashi®. These cells also produce substantial amounts of cytokines [63] and are involved in the body’s response to pathogen invasion, contributing to their elimination in oxygen-dependent and oxygen-independent processes. This is confirmed by the results of our research, in which high percentages of SWC3-expressing cells and cells participating in respiratory burst were noted in the experimental group, accompanied by a reduction in the severity of gastrointestinal and respiratory symptoms. It is worth noting that macrophages and polymorphonuclear cells do not reach full morphological and functional maturity until about 35 days of age [64], and chemotactic and phagocytic mechanisms are weak in young pigs [65]. Sudden environmental and dietary changes may be conducive to infection during this period [66], which adversely affects animal health. The use of probiotic preparations in pig feeding throughout the entire fattening period reduces this adverse effect by stimulating the activity of phagocytic cells [10]. Such observations were made in our experiment, which showed an increase in the percentage of SWC3-expressing cells and their phagocytic activity from the 21st day of life, indicating improvement in the body’s defense mechanisms. The results of numerous studies suggest that effective stimulation of phagocytic cells is conditioned by differences in the composition of the microbial cell wall, the presence of immunologically active peptides in it, and the ability of microorganisms to adhere to the intestinal mucosa [67, 68]. These phenomena are strictly dependent on the species or strain of bacteria included in the probiotic. Hence, the formula of the EM Bokashi® probiotic mixture used in our research enables effects at various stages of the immune response, which is expressed as better stimulation of the non-specific response conditioned by the large number of microbial strains that exert immunomodulatory effects and protect the body against excessive inflammation damaging the intestine.

The functions of peripheral phagocytes depend on the activation of integrin receptors, which condition an effective phagocytosis process [69]. Surface receptor integrin CD11b^+^/CD18^+^, also known as macrophage-1 antigen (Mac-1), complement receptor 3 (CR3), or αMβ2, is a receptor located on monocytes, macrophages, and neutrophils, and its main function is participation in phagocytosis. CD11b^+^/CD18^+^ also acts as an adhesion molecule on phagocytic cells [70]. In the present study, a significant increase in CD11b^+^/CD18^+^ expression in granulocytes and monocytes was shown in pigs receiving the EM Bokashi® preparation as compared to the control group between days 21 and 154 of the experiment. Similar observations were made by Hidemura et al. [71], who showed that the use of *Bifidobacterium longum* as a feed supplement in mice increased the expression of CD18 and CD62L receptors on polymorphonuclear cells, including phagocytic cells (neutrophils), which facilitates their migration to inflammation sites and the fight against infection. Similarly, the use of *L. lactis* in fish [72] significantly increased expression of the CR3 receptor on neutrophils, and therefore, this phenomenon can be used to monitor changes in the phagocytosis process due to immune system exposure to the antigens present in probiotics. In our study, the increased CR3 expression on granulocytes and monocytes induced by the EM Bokashi® preparation can therefore be regarded as stimulation of phagocytosis by the antigens contained in the preparation, which demonstrates that the non-specific immune response is stimulated by this probiotic. This phenomenon is one of the mechanisms by which the EM Bokashi® preparation enhances the defensive responses of pigs to the presence of pathogenic microbes. This seems to be confirmed by Vargas et al. 2015, who showed that the use of a probiotic preparation based on *L. rhamnosus* GG in poultry stimulates phagocytosis via the CR3 (CD11b/CD18) receptor present on most immune cells, and underlying this phenomenon is the direct interaction of the lactobacilli with macrophages [73]. The increased percentage of cells expressing SWC3 and CD11b^+^CD18^+^ and the increase in phagocytosis and respiratory burst demonstrated in our experiment should therefore be seen as the effect of contact of EM Bokashi® components with phagocytic cells within the GALT system. *Saccharomyces cerevisiae* present in many probiotic preparations, which contains β-glucan in the cell wall, exerts an additional strong stimulatory effect on the CR3 (CD11b/CD18) receptor located on neutrophils, monocytes, NK cells, and macrophages [74, 75]. The increase in the expression of this receptor following the use of the EM Bokashi® preparation in the present study can be linked to the effect of this receptor on immune processes providing defense against infection. Contrasting observations were made by Michałkiewicz et al., who showed that the use of a probiotic preparation containing *L. delbrueckii* ssp., *L. bulgaricus* LbY-27, *Bifidobacterium* BB-12 R, and *L. acidophilus* La-5R in the human diet resulted in inhibition of the expression of certain receptors on monocytes, including CD11b [76]. The fact that similar relationships were not demonstrated in our experiment can be explained by the diversity of the microbial strains contained in the EM Bokashi® preparation, which influence various immune mechanisms and together maintain homeostasis in the immune system.

It should be emphasized that strains of probiotic microorganisms, e.g., *Lactobacillus gasperi*, also produce chemotactic factors activating CD11b/CD18 on macrophages and other phagocytic cells [77]. It can therefore be assumed that the use of the probiotic EM Bokashi® formulation containing numerous microbial strains leads to the production of such factors by probiotic microorganisms in the gut, which stimulates non-specific immune mechanisms. This is evidenced by the increase in CD11b/CD18 expression and intensification of phagocytosis demonstrated in our study.

The experiment also showed a correlation between the percentage of phagocytic cells and that of granulocytes and monocytes displaying respiratory burst. A comparable increase in respiratory burst was demonstrated in the group of pigs receiving EM Bokashi® between days 21 and 70 and on day 154 of the study. The increased capacity to activate phagocytic cells in the group of pigs receiving the probiotic, manifested by the release of reactive oxygen species (ROS), is evidence of strong stimulation of the anti-infective response by EM Bokashi®. Similar observations were made by Calder and Kew, 2002 in a study on rats and mice, in which probiotics contributed to an increase in phagocytosis and respiratory burst in neutrophils [78]. Increased respiratory burst in monocytes and heterophils and increased proliferation of these cells following administration of a probiotic containing *Enterococcus faecium*, *Pediococcus acidilactici*, *Bifidobacterium animalis*, and *Lactobacillus reuteri* has also been demonstrated by Stringfellow et al. in a study on broilers [79]. The two phenomena resulted in stimulation of the immune response in birds vaccinated against coccidiosis. Similarly, Farnell et al., 2006 showed that oral administration of probiotics to poultry stimulated heterophil synthesis, oxidative burst, and degranulation of phagocytes, leading to stimulation of innate immunity and improving local immune mechanisms of the intestinal mucosa [80]. This phenomenon was also demonstrated in our experiment, in which oral administration of EM Bokashi® reduced the incidence of gastrointestinal disorders in pigs. Modulation of the immune response involving stimulation of respiratory burst has also been demonstrated in studies on humans [81] and pigs [82]. Zhu et al. [82] showed an increase in reactive oxygen species in the process of respiratory burst in post-weaning piglets. Excessive amounts of reactive oxygen species are known to damage DNA, proteins, and lipids, especially in the cell membrane, which manifests as impaired function of the intestinal barrier and diarrhea [83]. Although in our experiment, an increase in respiratory burst was observed on days 21 and 70, i.e., during the weaning period, in the group of pigs receiving the EM Bokashi® preparation, no increase in the incidence of piglets with symptoms of diarrhea was shown in this group. In addition, transient diarrhea was noted in only six pigs from the experimental group on day 28 of the study, while in the control group, 12 pigs had diarrhea symptoms on day 28, and 9 pigs on day 35 of the study. In addition to diarrhea symptoms, many animals in the control group also exhibited respiratory symptoms.

The bacterial strains included in the probiotic can induce production of TNF-α and IL-6, and significantly influenced on modulation of the body’s immune response by released cytokines [51, 84, 85]. This is confirmed by the results of the present study, which showed strong stimulation of synthesis of proinflammatory TNF-α between 35 and 154 days of the experiment in the pigs receiving EM Bokashi®. Such high TNF-α concentrations should be regarded as a reaction of PBMCs to the bacterial antigens contained in the probiotic. In addition, the use of probiotics with a diverse composition of microbes contributes to selective modulation of the native microbiota of the gastrointestinal tract, especially by increasing the concentration of lactic acid bacteria, which additionally increases the concentration of locally produced cytokines [86]. This phenomenon applies in particular to *S. bouldardii* in probiotics, which increases the synthesis and peripheral concentration of TNF-α while stimulating synthesis of IFN-γ [86], as confirmed in the present study. The high concentration of TNF-α in the experimental group is evidence of a Th1 cellular phenotype. The simultaneous increase in TNF-α concentration and phagocytic activity in the group of pigs receiving EM Bokashi® is confirmation of the activation of non-specific immune response mechanisms based on phagocyte activity. Similar observations were made by Franco et al., 2013 and Vinderola et al., 2005, who treated mice with kefir containing yeasts and acetic acid bacteria and found an increase in the percentage of phagocytic cells and an increase in production of TNF-α and IL-6 by macrophages [87, 88]. Similarly, in a study using fermented milk containing *Lactobacillus helveticus*, an increase was observed in TNF-α concentration, accompanied by higher NO production and increased phagocytic potential of phagocytes [89]. The increased TNF-α concentration in the experimental group may also indicate the activation of dendritic cells (DCs) in the intestinal lamina propria that release TNF-α [89, 90] and take part in recognition and elimination of the antigens contained in the EM Bokashi® preparation.

The increase in IL-6 concentration in the experimental group between days 63 and 154 of the study, in conjunction with the increase in the percentage of monocytes and granulocytes, indicates that this cytokine is produced by phagocytic cells in response to the bacterial antigens contained in the EM Bokashi® preparation, and perhaps, its role is to maintain a state of controlled inflammation. At the same time, an increase in the concentration of this cytokine increases the production of TNF-α through co-stimulation, as confirmed by the results obtained in the experimental group. The anti-inflammatory effect of IL-6, on the other hand, involved stimulation of IL-10 synthesis, which was also demonstrated in our experiment. The function of IL-10 in cellular immune surveillance is on the one hand a suppressive effect on the immune response, and on the other hand a modulating effect on cells with a Th profile [91]. IL-10 produced by macrophages and dendritic cells inhibits the synthesis and release of proinflammatory cytokines, including IL-1, TNFα, IL-6, and IL-12, by antigen-presenting cells, and inhibits T cell proliferation in response to antigens or superantigens [91]. The study showed that feed supplementation with EM increased the serum concentration of anti-inflammatory IL-10 in the pigs in the experimental group, which provides evidence of the dominance of the Th2 immune profile, a reduction in the severity of inflammation, support of the humoral immune response, and simultaneous inhibition of Th1 cytokine production.

The processes described are particularly important in the post-weaning period of pigs, during which, as shown by Pie et al. [92], the concentrations of pro-inflammatory cytokines IL-1b, IL-6, and TNF-α increase, which is linked to changes in the nature of antigens stimulating the intestinal epithelium, in feed composition, and in the living conditions of the animals. An increased IL-10 concentration, as in the experimental group in our study, has an inhibitory effect on the inflammatory response that can lead to enterocyte damage [93, 94]. It also ensures maintenance of immune tolerance to the diverse intestinal bacterial microbiota enriched with the EM Bokashi® preparation, preventing the development of allergic reactions in which macrophages participate as APC cells [95]. In the present study, the high phagocytic activity of phagocytes and respiratory burst in the experimental group, together with the high percentage of cells expressing SWC3 and CD11b/CD18^+^ and the profile of released cytokines, demonstrate maintenance of immune balance between the pro- and anti-inflammatory response, which determines homeostasis in the body.
